# In this issue

**Published:** 2022-10

**Adipose-derived mesenchymal stem cells and wound healing. *Potential clinical applications in wound repair***

Almalki discusses and review the therapeutic potential of AMSCs for wound repair via acceleration of wound closure, re-epithelialization, enhancement of angiogenesis and immunomodulation of prolonged inflammatory responses, as well as the current challenges in clinical implementation. He concluded that AMSC-based therapy for non-healing or chronic cutaneous wounds in animal models has been shown to accelerate wound healing through different mechanisms. Therefore, the use of autologous AMSCs to promote cutaneous wound healing in patients appears to be a promising therapeutic strategy.

***see page 1075***

**Figure:**
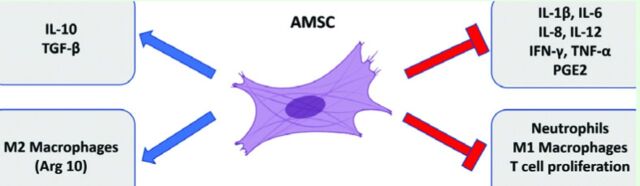
Mesenchymal stem cells immunomodulatory roles in wound closure.

