# Supplementary material for: In this issue
Source: Saudi Med J. 2022 Oct;43(10):1073. (PMC9994495)
Supplement: Related texts

# Abstract: Original Articles

**Evaluation of diaphragm thickness to predict intubation requirement and mortality in critical COVID-19 patients**

Dal et al investigate the value of measuring the DT on thorax CT at ICU admission for predicting intubation requirement and mortality among COVID-19 patients. The patients’ demographic characteristics, laboratory parameters, DT measurements, MV requirements, and mortality statuses are retrospectively screened. The relationships between DT on initial CT, MV requirement, and mortality are investigated. They concluded that mortality and MV requirements can be predicted from DT measurements. Diaphragm thickness can facilitate the identification of high-risk patients on CT evaluation at ICU admission.

***see page 1120***

**Figure:** Points of diaphragm thickness measurements.

**Self-esteem mediates the relationship perceived stigma with self-efficacy for diabetes management in individuals with type 2 diabetes mellitus**

Ozturk et al determine the mediating effect of self-esteem in the relationship between the perceived stigmatization of individuals with T2DM and their self-efficacy regarding diabetes management. A descriptive information form, diabetes management self-efficacy scale, Rosenberg self-esteem scale, and type-2 diabetes stigma assessment scale are used in data collection. They concluded that minimizing the perceived stigmatization can improve patients’ diabetes management self-efficacy. With patient training programs and individualized nursing care plans prepared by psychiatric nurses to provide psychological support patients and through their interventions that increase self-esteem, self-stigmatization can be reduced.

***see page 1157***

# Abstract: Case Report

**Post COVID-19 pulmonary mass. *Case report and review of literatures***

Hussen present an unusual and a rare pulmonary affection by COVID-19, in which only one lung is affected. Coronavirus disease-19 attacks the lungs and interferes seriously with their functions. The attack is usually bilaterally, while a uni lateral pulmonary affection is unusual. The presentation, both clinical and radiological findings, bronchoscopy appearance, the strange operative findings of the resected mass, the uneventful post-operative course, in addition to the histopathological report, is presented. He concluded that unilateral lung affection is unusual and post-viral pneumonia COVID-19 should be considered as a possible aftermath, which may not be uncommon in Iraq.

***see page 1165***

**Figure:** An operative view of the mass.
